# Immunohistochemical Investigation of Cyclooxygenase-2 Expression in Rabbit Uterine Adenocarcinoma and the Potential Use of COX-2 Inhibitors in Cancer Therapy

**DOI:** 10.3390/ani14223169

**Published:** 2024-11-06

**Authors:** Emanuela Vaccaro, Luigi Navas, Martina Ercolano, Giuseppe Piegari, Evaristo Di Napoli, Serenella Papparella, Donato Inverso, Barbara Brunetti, Orlando Paciello, Valeria Russo

**Affiliations:** 1 Department of Veterinary Medicine and Animal Productions, University of Naples Federico II, 80137 Naples, Italy; emanuela.vaccaro@unina.it (E.V.); lnavas@unina.it (L.N.); martinaercolano@gmail.com (M.E.); papparel@unina.it (S.P.); paciello@unina.it (O.P.); valeria.russo@unina.it (V.R.); 2 Vascular Pathobiology Unit, San Raffaele Scientific Institute, 20132 Milan, Italy; inverso.donato@hsr.it; 3 Department of Veterinary Medical Sciences, University of Bologna, Ozzano dell’Emilia, 40126 Bologna, Italy; b.brunetti@unibo.it

**Keywords:** rabbit, endometrial adenocarcinoma, COX-2, immunohistochemistry, meloxicam

## Abstract

Cyclooxygenase-2 (COX-2) is overexpressed in many human and animal cancers. Selective COX-2 inhibitors have shown antitumoral effects in tumors with a high expression of COX-2. This study evaluates (1) the expression of COX-2 in rabbit uterine adenocarcinomas, (2) the correlation between immunophenotypic expression and histopathological changes, and (3) the post-surgery response to therapy with COX-2 inhibitors. Forty rabbit uteri were divided into three groups: *neoplastic*, *hyperplastic*, and *normal endometrium*. A histological and immunohistochemical score was applied to investigate the tumor’s grade and the COX-2 expression. By histological evaluation, 30 cases of endometrial adenocarcinoma, 5 cases of endometrial hyperplasia and 5 normal endometria were found. Of the six cases of endometrial adenocarcinoma with follow-up available, four received a post-surgical treatment with meloxicam and two were treated by surgery alone. The survival time of the animals treated with meloxicam was longer than that observed in the untreated animals. A statistically significant difference in COX-2 IHS was observed between non-neoplastic endometrium and adenocarcinoma. The progressive increase in COX-2 expression from normal epithelium to carcinoma suggests that upregulation of COX-2 expression may play a role in tumor initiation and progression. Our findings suggest the possible use of COX-2 inhibitors in treating uterine adenocarcinoma in rabbits. Further study will be needed to confirm this hypothesis.

## Simple Summary

Uterine adenocarcinoma is the most common type of cancer seen in rabbits. The enzyme cyclooxygenase-2 (COX-2) is overexpressed in several cancers and has been implicated in the process of carcinogenesis. Selective COX-2 inhibitors have shown antitumoral effects in different cancers with high expressions of COX-2. In the literature, there are no studies about the expression of COX-2 in rabbit uterine adenocarcinoma. We investigated the expression of COX-2 in uterine endometrial cancer and the possible use of meloxicam in cancer therapy in rabbits. COX-2 expression was determined by immunohistochemistry in 30 cases of endometrial adenocarcinoma, 5 cases of endometrial hyperplasia, and 5 normal control cases. COX-2 was found to be overexpressed in all cases of uterine adenocarcinoma examined. The survival time of the animals treated with meloxicam was longer than that observed in the untreated animals. In conclusion, these results suggest a role for COX-2 in the development and progression of endometrial carcinoma and the possible use of COX-2 inhibitors in cancer treatment in rabbits.

## 1. Introduction

Uterine adenocarcinoma is a malignant tumor of the uterus that arises from the secretory tissue and is the most common type of neoplasm seen in rabbits [1,2] with an incidence of up to 80% in females older than five years [2]. The incidence of this tumor increases with age. Age-related disorders in the endometrium may promote tumor formation, but no correlation has been demonstrated between the prevalence of uterine tumors in rabbits and their breeding history. Peritoneal metastases may develop if the tumor infiltrates the uterine wall and distant metastases can be found, especially in the lungs, liver, bones, brain, and skin [3,4,5,6]. The treatment of choice is ovariohysterectomy in clinically stable patients with periodic follow-ups recommended to monitor metastases. There are no other treatments currently available [3,6]. 

### 1.1. Cyclooxygenase

Cyclooxygenase (COX) is responsible for converting arachidonic acid to prostaglandins (PGs). Cyclooxygenase-derived proteinoids contribute to many normal physiologic processes including hemostasis, platelet aggregation, kidney and gastric function, reproduction, pain, and fever. Cyclooxygenase has two isoforms, which are COX-1 and COX-2. COX-2 is an inducible enzyme present during inflammatory processes in inflammation-affected tissues. Many studies demonstrated that COX-2 is essential for postnatal development and multiple female reproductive processes [7,8]. COX-2 has also been associated with carcinogenesis. Recent studies suggest that neoplastic transformation may result from deregulating critical steps in the epidermal growth factor receptor signaling pathway, including the Ras signaling pathway [9,10]. According to the literature, COX-2 is overexpressed in several human cancers, like those of the colon, lung, breast, pancreas, esophagus, urinary bladder, squamous cell carcinoma of the head and neck and uterine endometrial carcinoma [11,12,13,14]. The mechanisms by which COX-2 participates in oncogenesis are complex and poorly understood in animals. Studies indicate that the overexpression of COX-2 may be necessary for tumor invasion, angiogenesis, and resistance to apoptosis [15]. Considering these pieces of evidence, understanding COX dynamics in different neoplastic contexts with a comparative pathology approach promises to provide fundamental insights into defining novel therapeutic approaches targeting COX activity. In dogs, cats, and humans, COX-2 is overexpressed in many types of tumors [16,17]. It seems that epithelial neoplasms are inclined to express high levels of the inducible form of cyclooxygenase [16]. Many immunohistochemical studies have also demonstrated the overexpression of COX-2 in human endometrial tumors [13,14]. COX-2 expression was significantly more frequent in endometrial cancer (53.1% of cases) than in normal endometrium in the proliferative or secretory phase (10% of cases) [13]. However, data on its prognostic significance and whether its expression is a late or an early step in developing endometrial carcinogenesis are unclear [14]. In dogs, the expression of COX-2 has been reported in many types of cancers, such as mammary tumors, prostatic carcinoma, transitional cell carcinoma, squamous cell carcinoma, intestinal epithelial tumors, nasal epithelial tumor, renal cell carcinoma, oral melanomas, osteosarcomas, and meningiomas [17]. In cats, the expression of COX-2 has been reported in different types of tumors, such as squamous cell carcinoma, mammary carcinoma, pulmonary adenocarcinomas, intestinal adenocarcinomas, lymphomas, and vaccine-associated sarcomas [16].

### 1.2. Role of Selective COX-2 Inhibitors

The association between the expression of COX-2 and tumors led to new research based on the possibility of therapies using COX-2 inhibitors. COX-2 is targeted for various non-steroidal anti-inflammatory inhibitors (NSAIDs) [7]. COX-2 inhibitors have been found to have chemo-preventive and antitumor effects and potentiate chemotherapy’s effects [7,18]. Treatment with COX-2 inhibitors has been studied in many human cancers [19,20,21,22]. In animals, therapy with COX-2 inhibitors reduces the incidence and growth of tumors [23,24]. Studies suggest that tumor response to anti-COX-2 drugs was correlated to apoptosis induction and angiogenic growth factor reduction. The mechanisms by which COX inhibitors exert their antitumor effects are not entirely understood; it has been shown to correlate to the decrease in COX products such as PGE2, which are implicated in tumor cell resistance to apoptosis and induction of angiogenesis [18]. Meloxicam is a non-steroidal anti-inflammatory drug used in veterinary medicine [25]. The therapeutic effects of meloxicam have already been confirmed in both in vitro and in vivo studies [24,26]. Several studies suggest a correlation between the overexpression of COX-2 and poor prognosis. COX-2 overexpression has been associated with increased metastatic potential with activation of matrix-degrading metalloproteinase enzymes, and in human endometrial cancer, has also been associated with myometrial invasion and the histological grade of the tumor [27].

### 1.3. The Aim of the Study

No studies are available in the literature about the expression of COX-2 and the use of COX-2 inhibitors in rabbit uterine adenocarcinoma. In light of these observations, the aims of this study are: (1) to evaluate the expression of COX-2 in rabbit uterine adenocarcinomas; (2) to investigate the correlation between immunophenotypic expression and histopathological features; (3) to assess post-surgery response to therapy with a COX-2 inhibitor.

## 2. Materials and Methods

### 2.1. Study Overview and Case Selection

Tissue blocks of formalin-fixed paraffin-embedded tissues of 40 uteri from rabbits were selected from the histological archives of the Department of Veterinary Medicine and Animal Productions of the University of Naples Federico II between 2016 and 2023. The submission forms and histological reports were collected to obtain information about the age of the animals and the histological diagnosis. Based on the histological diagnosis, tissue samples were divided into 3 groups: Group A: neoplastic; Group B: hyperplastic; and Group C: normal endometrium. Where available, follow-up data of the animals with a histological diagnosis of neoplasia were extracted from the informatic system of the Department of Veterinary Medicine and Animal Production of the University of Naples “Federico II” (MyClinical). The animals were divided based on whether they were treated with surgery alone or with the COX-2 inhibitor (meloxicam 0.5 mg/kg twice daily for 7 days) after surgical excision. Information on survival time and postoperative diagnosis of metastasis was also evaluated.

### 2.2. Histopathological Examination

Group A histological samples were evaluated to investigate growth patterns and biological behavior. Specifically, mitotic count, invasion of the myometrium, and angio- or lymph angioinvasion were assessed for each case. The number of mitoses was counted per 10 high power (HP) fields (×10 ocular, ×40 objective). The myometrium invasion was classified as a superficial invasion of the myometrium (less than 50%) and a deep invasion of the myometer (more than 50%) [28,29]. Furthermore, the histological grade was defined using a scoring system previously reported by the International Federation of Gynecology and Obstetrics (FIGO). The histological samples were graded as follows: Grade 1 (G1), 5% or less of tumor tissue is solid tumor growth, and the neoplastic cells are well differentiated; Grade 2 (G2), 6–50% of the tissue is solid tumor growth, and the neoplastic cells are moderately differentiated; and Grade 3 (G3), more than 50% of the tissue is solid tumor growth, and the neoplastic cells are poorly differentiated [30]. Finally, groups B and C were evaluated to investigate the morphological alterations.

### 2.3. Immunohistochemistry

Uterine tissue sections were deparaffinized and rehydrated with a decreasing series of alcohol, while the endogenous peroxidase activity was blocked by incubation in 0.3% H_2_O_2_ in methanol for 15 min. Antigen retrieval was performed by pretreating via microwave heating in a citrate buffer pH 6.00. The slides were washed with phosphate-buffered saline (PBS, pH 7.4, 0.01 M), then incubated for 1 h at room temperature with normal goat serum (Santa Cruz Biotechnology, 10410 Finnell St, Dallas, TX, USA) diluted at 20% in PBS. A polyclonal mouse anti-cyclooxygenase 2 primary antibody (clone ab88522, Abcam, Cambridge, UK) diluted at 1:50 in PBS was applied overnight at +4 °C. The slides were washed with PBS, then incubated for 30 min with a goat anti-mouse biotinylated secondary antibody (Vector Laboratories Inc., Newark, CA, USA) diluted at 1:200 in PBS. Then, the sections were incubated with Vectastain ABC reagent (Vector Laboratories Inc., Newark, CA, USA) for 30 min at room temperature. Color development was obtained by treatment with 3,3′-diaminobenzidine (Vector Laboratories Inc., 6737 Mowry Ave, Newark, CA, USA) and then counterstained with Carazzi’s hematoxylin. Negative control tissues were treated similarly except that normal non-immune mouse serum was substituted for the COX-2 antibody. Rabbit uteri with inflammation and sections of rabbit corpora lutea were used as positive control tissues [31]. For each case, ten 20× fields were randomly photographed with Pannoramic scan II (3 Dhistech, The Digital Pathology Company, Budapest Öv u. 3 1141, Budapest, Hungary), and each photo was elaborated with Fiji (ImageJ2. 14. 0, National Institutes of Health, Bethesda, MD, USA) to quantify COX-2 expression in each sample.

### 2.4. Immunohistochemical Score System

Quantitative and semiquantitative staining scores were used to evaluate epithelial and stromal immunolabelling for COX-2. In particular, the quantity score was assessed as follows: 0 = no staining; 1 = 1–10% immunopositive cells; 2 = 11–50% immunopositive cells; 3 = 51–80% immunopositive cells; 4 = ≥81% immunopositive cells. COX-2 staining intensity was instead evaluated as follows: 0 = no staining 1 = weak staining 2 = moderate staining 3 = strong staining. The intracellular staining patterns were evaluated as cytoplasmic. Ten fields at 20× magnification were assessed for each section by two independent pathologists (E.V. and V.R.) with a concordance rate of 97%. Finally, the COX-2 immunohistochemical score (COX-2 IHS) was obtained by multiplying the quantitative score and staining intensity score and then classified as negative or weak (0–3), moderate (4–8), or strong (9–12) [32].

### 2.5. Statistical Analysis

The SPSS 20.0 package (SPSS Inc., Chicago, IL, USA) was used for statistical data analysis. The Mann–Whitney U test, a nonparametric test for two independent samples, was used to assess the differences in COX expression between groups (Group A: neoplastic; Group B: hyperplastic; and Group C: normal endometrium). The positive or negative associations between the mitotic count, myometrial invasion, histological grade, and COX expression in Group A cases were evaluated by Spearman’s rho correlation; *p* < 0.05 was considered significant.

## 3. Results

### 3.1. Rabbit Demographics and Histological Diagnosis

Out of the 40 examined reports, we found 30 cases of endometrial adenocarcinoma (Group A; 75%), 5 cases of endometrial hyperplasia (Group B; 12.5%), and 5 normal endometria collected from rabbits undergoing routine spay surgeries (Group C; 12.5%). The mean age of animals with a histopathological diagnosis of neoplasia was 4.8 years (age range: 1–10; median: 4), the mean age of animals with endometrial hyperplasia was 3 (age range: 1–9; median: 2) while the mean age of rabbit with normal endometrium was 7.5 months (age range: 6–10 months; median: 7 months). The selected cases represented six breeds, including mixed breed rabbit (*n* = 16, 40%), Aries (*n* = 6, 15%), Dwarf Aries (*n* = 3, 7.5%), Dwarf (*n* = 5, 12.5%), and Lionhead (*n* = 8, 20%), Giant (*n* = 2, 5%).

### 3.2. Histopathological Evaluation

**Figure 1:**
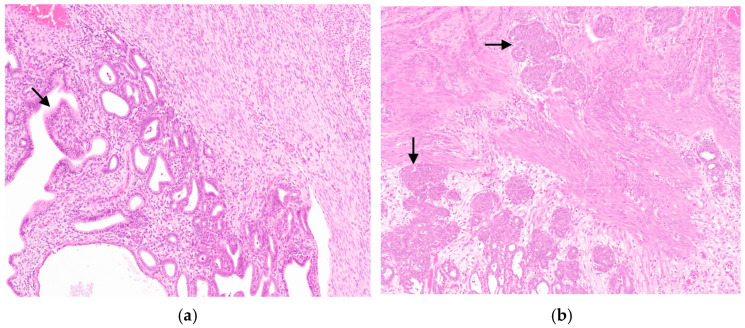
Histological sections of rabbit uterine adenocarcinoma: (**a**) Papillary adenocarcinoma; neoplastic cells commonly show protrusion into the lumina of the neoplastic glands (arrow). (**b**) Tubular/solid adenocarcinoma; neoplastic glands infiltrate into the myometrium (arrows) without thinning of the myometrium and endometrial hyperplasia. Hematoxylin and eosin (original magnification 200×).

Group A. There were thirty uterine adenocarcinomas classified based on growth pattern: nine of the papillary subtype and twenty-one of the tubular/solid subtype. In papillary adenocarcinoma, there were several papillary projections within the glandular lumen and a loss of much of the surrounding stroma (Figure 1a). In contrast, the tubular/solid subtype is characterized by tubules, nests, acini, and solid cellular regions (Figure 1b).

Neoplastic cells often form tubular structures in the lumen (Figure 1b).

The myometrial invasion was seen in all adenocarcinomas, in particular, 10 cases with superficial invasion and 20 with deep invasion. The mitoses were <1 for 10HPFs in 4 cases, 1–5 for 10HPFs in 12 cases, and >5 for 10HPFs in 14 cases. No vascular/lymphatic invasion was observed (Table 1).

**Figure 2:**
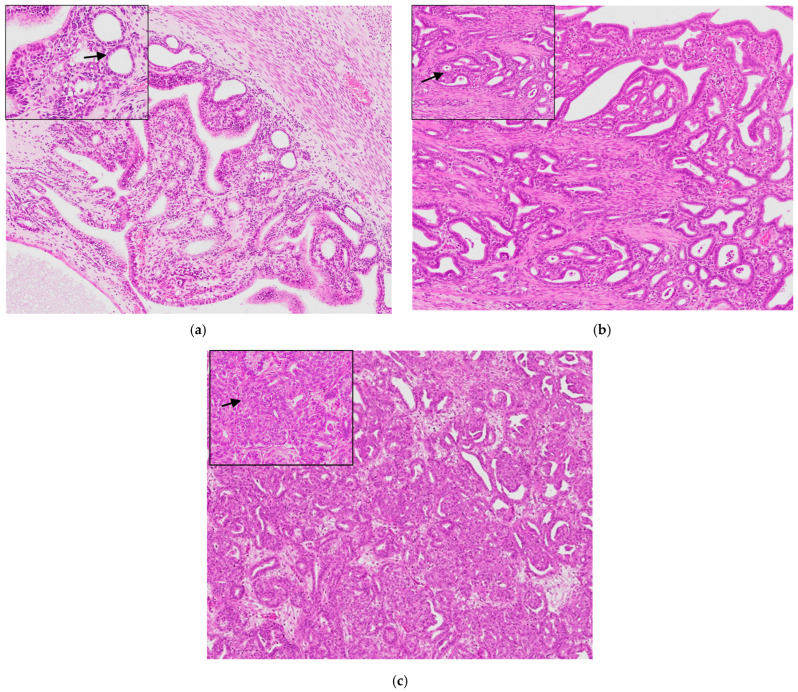
The histopathological evaluation of endometrial adenocarcinoma: (**a**) Grade 1. Inset: the tumor cells are well differentiated (arrow); (**b**) Grade 2. Inset: the tumor cells are moderately differentiated (arrow); (**c**) Grade 3. Inset: the tumor cells are poorly differentiated, and most tissue is solid tumor growth (arrow). Hematoxylin and Eosin (original magnification 200× and high magnification 400×).

Based on the FIGO system, uterine adenocarcinomas have been classified into three grades: 5 were Grade 1 (17%), 8 were Grade 2 (27%), and 17 were Grade 3 (56%). Grade 1 adenocarcinoma displayed well-differentiated tumor cells with eosinophilic cytoplasm without solid growth. Rare mitoses were observed. The myometrial invasion was not evident (Figure 2a). In Grade 2, the tumor cells and tissue with limited solid growth were moderately differentiated. Randomly distributed areas of necrosis within the tumors were commonly observed (Figure 2b). In Grade 3, the endometrium is extensively involved by an ill-defined, non-encapsulated, infiltrating neoplasm, forming small-caliber tubules, papillae, and solid nests associated with scant fibrovascular stroma. The neoplastic cells are polygonal, with homogeneous and eosinophilic cytoplasm. The nuclei are paracentral with marginated chromatin and prominent single nucleoli. Moderate anisokaryosis and anisocytosis are observed. Mitoses are numerous (Figure 2c).

**Figure 3:**
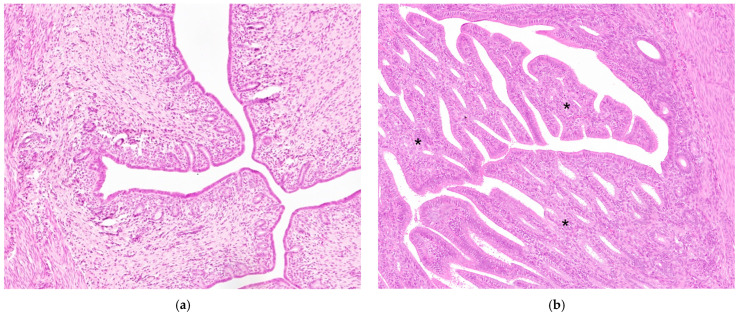
Histological sections of normal endometrium (**a**) and endometrial hyperplasia, characterized by exophytic papillary projections (asterisks) (**b**). Hematoxylin and eosin (original magnification 200×).

Group B: Endometrial hyperplasia exhibited marked and irregular thickening characterized by exophytic papillary projections and glands with dilated lumen bordered by a row of columnar cells with sparse pleomorphism (Figure 3b).

Group C: Normal endometrium showed a mild inflammatory infiltrate characterized mainly by macrophages and lymphocytes.

### 3.3. Immunohistochemistry

**Figure 4:**
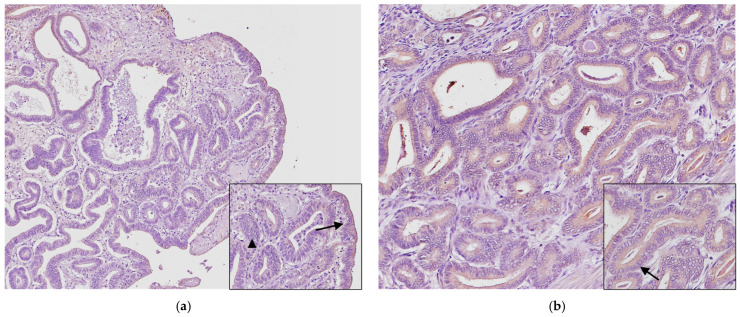

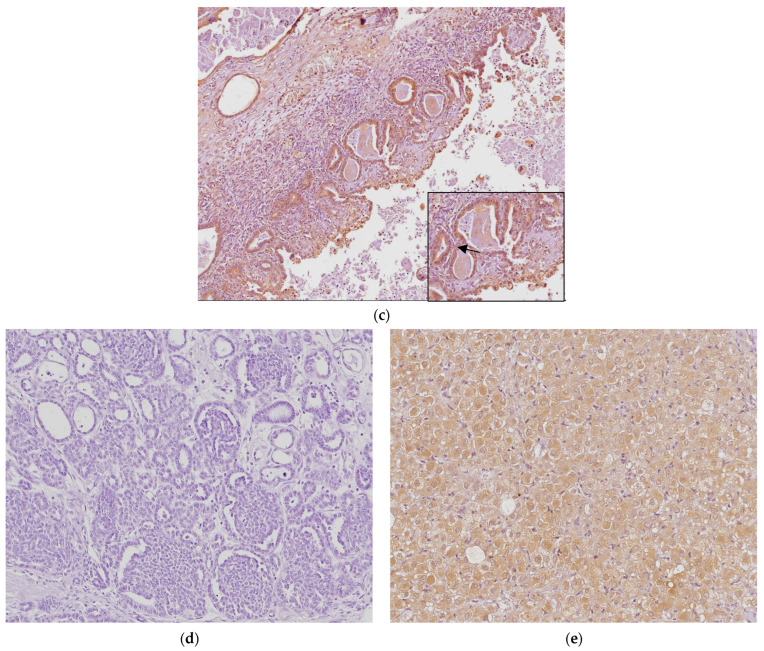
COX-2 expression in uterine adenocarcinoma. Epithelial tumor cells show granular and diffuse cytoplasmatic staining: (**a**) weak staining, (**b**) moderate staining, and (**c**) intense staining. (**d**) Negative control: no immunoreactivity was detected when the primary anti-COX-2 antibody was replaced by normal non-immune mouse serum. (**e**) Positive control: sections of rabbit corpora lutea. Avidin-biotin-peroxidase complex method, hematoxylin counterstain (original magnification 200×). Inset (**a**–**c**): weak to strong COX-2 immunoreactivity in the cytoplasm of epithelial tumor cells (arrows) and immunonegative tumor cells (arrowhead) (high magnification 400×).

Group A. In neoplastic tissues, COX-2 expression was manifested as a cytoplasmic diffuse and a granular pattern of immunoreactivity in epithelial tumor cells, only focal weak staining in stromal cells was evident (Figure 4) and in immune cells present in tumors with inflammation. The uterine adenocarcinomas presented different COX-2 immunohistochemical scores (COX-2 IHS). COX-2 IHS was weak in 15 cases (47%) (Figure 4a), 12 cases (43%) were moderate (Figure 4b), and 3 cases (10%) were strong (Figure 4c) (Table 2).

**Figure 5:**
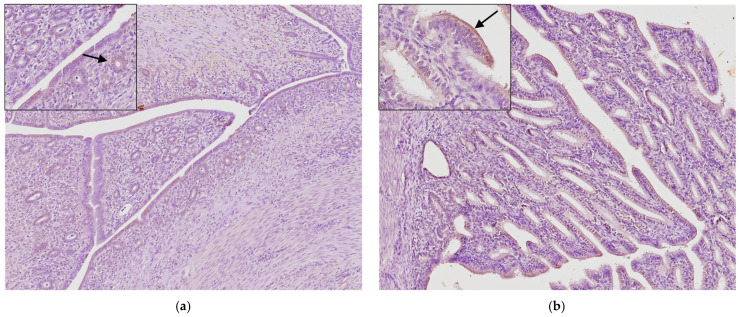
COX-2 immunostaining uterine tissue section. Normal endometrium: weak cytoplasmatic immunoreactivity for COX-2 was observed in the epithelial cells: (**a**). Endometrial hyperplasia: epithelial cells show weak cytoplasmic staining; (**b**) (original magnification 200×). Inset: epithelial cell with weak cytoplasmatic intensity (arrows) (high magnification 400×).

Group B: A weak cytoplasmatic immunoreactivity was evident in the epithelium of endometrial hyperplasia. COX-2 IHS was weak (Figure 5b and Table 3).

Group C: In normal endometrium, COX-2 expression was weakly detected in the endometrium’s epithelium and the glandular epithelium’s cytoplasm. The COX-2 IHS was weak, and in only one case it was negative (Figure 5a and Table 4).

### 3.4. Follow-Up and Therapy

Follow-up data were available in 6 of 30 cases of adenocarcinoma. Of these, two rabbits were treated with surgery alone and four with meloxicam after surgical excision. No metastasis was observed in all rabbits treated with meloxicam until 18 months after surgery. Of these four rabbits, three were diagnosed with Grade 2 adenocarcinoma and papillary subtype and one with Grade 3 and tubular/solid subtype. In contrast, two untreated rabbits died with suspected lung metastasis between 4 and 12 months after surgical excision, and both had Grade 3, tubular/solid adenocarcinoma. The survival time of meloxicam-treated animals (>18 months) was longer than that observed in animals treated with surgery alone (mean: 5.4 months) (Table S1).

### 3.5. Statistical Analysis

**Figure 6:**
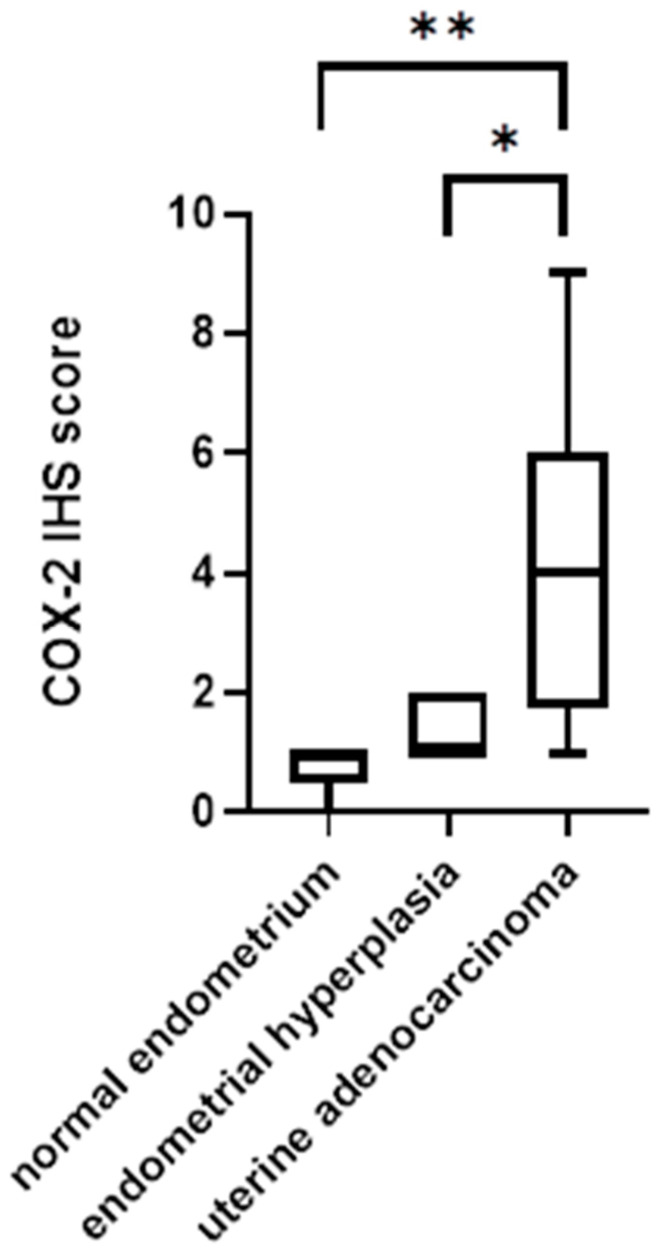
Statistical evaluation of the COX-2 IHS score in normal endometrial, endometrial hyperplasia, and uterine adenocarcinoma. The Mann–Whitney test showed a statistically significant difference among the assessed group (* *p* < 0.05, ** *p* < 0.01) except between normal endometrium and endometrial hyperplasia.

Statistical analyses showed no statistically significant difference between COX-2 immunohistochemical scores (COX-2 IHS) and tumor histologic grade (*p* = 0.0943) and between tumor histologic grade and mitotic count (*p* = 0.6679). The myometrial invasion was significantly different compared with histologic grade and COX-2 IHS (*p* < 0.0001; *p* < 0.0011). Furthermore, a statistically significant difference was observed between histological subtype and mitotic count (*p* < 0.05) and between histological grade (FIGO) and subtype (*p* < 0.0001). However, a statistically significant difference in COX-2 IHS was demonstrated between non-neoplastic endometrium and endometrial hyperplasia compared with uterine adenocarcinoma (* *p* < 0.05, ** *p* < 0.01) (Figure 6).

## 4. Discussion

In the present study, we investigated the morphological features and COX expression of both neoplastic and non-neoplastic rabbit uterine samples. In total, 30 cases of endometrial adenocarcinoma (Group A; 75%), 5 cases of endometrial hyperplasia (Group B; 12.5%), and 5 normal endometria (Group C; 12.5%) were investigated. Uterine adenocarcinomas were classified into papillary and tubular/solid subtypes based on growth pattern and according to the classification system reported by Asakawa et al. [28]. Furthermore, they were graded using a scoring system previously reported by the International Federation of Gynecology and Obstetrics (FIGO). Specifically, the classification system described by Asakawa et al. [28] is currently valid for histopathological evaluation of uterine carcinoma in rabbits; in contrast, the FIGO system finds application for endometrial cancer evaluation in human medicine. However, due to the similarity between rabbit and human cancer, it is also applied in our study. Indeed, in both species, there are signs indicating relationships between endometrial carcinomas and sex hormones, especially estrogens [33]. In addition, they show similar histologic features, such as the architecture consisting of confluent or back-to-back glands lacking intermediate stroma, cribriform or microacinar configurations, and a complex papillary, micropapillary, or villoglandular pattern; all these characteristics make the rabbit an ideal animal model for human endometrioid adenocarcinoma and justify the application of a human score system in the evaluation of rabbit adenocarcinoma in our study [34]. According to Asakawa et al., myometrial invasion was seen in all studied adenocarcinomas. It was superficial in 10 cases and deep in the remaining 20 cases. Furthermore, mitotic count ranged between 4 and 14 for 10 high-power fields in all cases assessed. Based on the FIGO system, 5 uterine adenocarcinomas were classified as Grade 1 (17%), 8 were classified as Grade 2 (27%), and 17 were classified as Grade 3 (56%). Spearman’s rho test showed a positive significant correlation between mitotic count and histological grade (Rho = 0.688; *p* < 0.01; FIGO system) and a negative correlation between mitotic count and COX expression. However, no statistical correlation was observed between histological grade and COX expression. These findings differ from those observed in humans, in which COX is strongly associated with the degree of differentiation of endometrial carcinomas. Overall, COX-2 plays a key role in the endometrium homeostasis in humans. Indeed, it is constitutively expressed in normal tissues and appears essential for blastocyst implantation and decidualization [35]. Several factors, such as estrogen, hypoxia, proinflammatory cytokines, environmental pollutants, metabolites, metabolic enzymes, and platelets, can regulate COX-2 expression. Furthermore, high concentrations of COX-2 lead to cell proliferation, low levels of apoptosis, invasion activity, angiogenesis, and infertility [36]. In animal endometrial tumors, the expression of COX-2 has still not been explored. As in humans, COX-2 is overexpressed in many types of tumors in dogs, cats, and horses. However, the exact role of COX-2 in the oncogenesis of tumors in different animal species is still poorly understood [17]. In the bitch, COX-2 expression is correlated with inflammatory conditions [37]. Instead, in feline, sheep, and pigs, COX-2 epithelial expression varies according to the estrous cycle stage [31]. To our knowledge, no studies on COX-2 expression in rabbits under pathophysiological conditions exist. In our study, the Mann–Whitney U test showed a statistically lower expression of COX-2 in hyperplastic uterine samples (Group B) compared with neoplastic (Group A) and a lower degree of COX expression in normal uterine samples (Group C) than that observed in hyperplastic uterine samples (Group B). In veterinary medicine, there is little heterogeneous information on the prognostic significance of COX-2 expression in the uterus [38,39]. However, the progressive increase in COX-2 expression from normal epithelium through rabbit endometrial hyperplasia and carcinoma suggests that upregulation of COX-2 expression may play a role in tumor onset and progression. Indeed, several studies have reported that in women, endometrial hyperplasia is associated with a higher risk for progression to endometrial carcinoma [40]. However, this correlation is still controversial in rabbits. Indeed, some authors report a possible correlation between hyperplasia and adenocarcinoma [33,41,42,43], while others describe no correlation [28,44]. Regarding COX expression, our findings are consistent with those reported in women in which the COX expression is upregulated in uterine cancer. In contrast, differences in COX expression patterns between normal, hyperplastic, and neoplastic uteri have not been observed in other species such as cats. Our findings suggest a different prognostic role of both endometrial hyperplasia and COX expression between species. Furthermore, our data highlight the importance of further investigating the pathological mechanism of adenocarcinoma development in rabbits.

Finally, the upregulation of COX in rabbit adenocarcinoma observed in our study suggests a possible therapeutic role of COX-2 selective inhibitors in rabbit cancer therapy. This hypothesis can be partially supported by the follow-up information evaluated in our study. Indeed, despite the small number of studied cases (four rabbits treated with meloxicam and two untreated), a longer survival time was observed in rabbits treated with meloxicam than the untreated rabbits. The therapeutic role of meloxicam in this species could result from the inhibition of COX-induced products, such as PGE2 [18]. However, further investigations, including a larger series of cases with a longer follow-up period, will be needed to confirm this hypothesis.

## 5. Conclusions

The upregulation of COX-2 in hyperplastic and neoplastic tissues observed in our study suggests its involvement in cancer development and progression in rabbits. Our preliminary results also suggest a possible use of COX inhibitors as therapeutic tools in rabbits. However, further investigations, including a more extensive series of cases with a longer follow-up, will be needed to confirm this hypothesis.

## Figures and Tables

**Table 1:** Histological features of rabbit endometrial adenocarcinomas.

	Number of Cases
**Adenocarcinoma**	
Papillary	9
Tubular/solid	21
**Myometrial invasion**	
Superficial	10
Deep	20
**Vascular/lymphatic invasion**	0
**Mitotic count (×10 HPFs)**	
≤1	4
1–5	12
x ≥ 5	14

**Table 2:** COX-2 IHS in uterine adenocarcinomas.

Case Number	Histologic Grade	COX-2 Quantity Score	COX-2 Intensity Score	Cox-2 IHS
1	1	2	2	4
2	3	3	2	6
3	3	1	1	1
4	3	1	2	2
5	3	1	1	1
6	3	1	1	1
7	3	3	3	9
8	3	2	2	4
9	3	1	1	1
10	1	1	1	1
11	1	2	3	6
12	2	2	1	2
13	3	2	1	2
14	2	2	3	6
15	3	2	2	4
16	3	2	1	2
17	2	3	2	6
18	3	2	2	4
19	2	2	1	2
20	2	2	2	4
21	2	2	2	4
22	3	3	3	9
23	3	1	1	1
24	1	2	2	4
25	3	3	3	9
26	2	2	1	2
27	3	3	2	6
28	3	2	1	2
29	2	1	1	1
30	1	2	1	2

**Table 3:** COX-2 IHS in endometrial hyperplasia.

Case Number	COX-2 Quantity Score	COX-2 Intensity Score	Cox-2 IHS
1	1	2	2
2	1	1	1
3	1	1	1
4	1	1	1
5	1	2	2

**Table 4:** COX-2 IHS in normal endometrium.

Case Number	COX-2 Quantity Score	COX-2 Intensity Score	Cox-2 IHS
1	1	1	1
2	1	1	1
3	1	1	1
4	0	0	0
5	1	1	1

## Data Availability

All relevant data are listed in the manuscript.

## Supplementary Materials

The following supporting information can be downloaded at: https://www.mdpi.com/article/10.3390/ani14223169/s1, Table S1: Information about the 6 cases with follow-up.
