# Supplementary material for: Immunohistochemical Investigation of Cyclooxygenase-2 Expression in Rabbit Uterine Adenocarcinoma and the Potential Use of COX-2 Inhibitors in Cancer Therapy
Source: Animals (Basel). 2024 Nov 6;14(22):3169. doi: 10.3390/ani14223169 (PMC11590897; doi:10.3390/ani14223169)
Supplement: Supplementary file 1 [file animals-14-03169-s001.zip › animals-3276838-supplementary.pdf]

Table S1. Information about the 6 cases with follow-up.

| Case | Histologic grade | Histologic subtype | Cox-2 IHS score | Therapy | Survival/Alive |
|---|---|---|---|---|---|
| 2 | 3 | Tubular/solid | 6 | meloxicam | yes |
| 16 | 3 | Tubular/solid | 2 | no | Dead (suspected lung metastasis) |
| 18 | 3 | Tubular/solid | 2 | no | Dead (suspected lung metastasis) |
| 17 | 2 | Papillary | 3 | meloxicam | Yes |
| 19 | 2 | Papillary | 2 | meloxicam | Yes |
| 20 | 2 | Papillary | 2 | meloxicam | yes |
